# Patients’ experiences of eHealth in palliative care: an integrative review

**DOI:** 10.1186/s12904-020-00667-1

**Published:** 2020-10-14

**Authors:** Cecilia Widberg, Birgitta Wiklund, Anna Klarare

**Affiliations:** 1 Stockholm Sjukhem Foundation, Department of Palliative Care, Stockholm, Sweden; 2 Department of Health Care Sciences, Palliative Care Research Centre, Ersta Sköndal Bräcke University College, Box 11189, SE-100 61 Stockholm, Sweden; 3 Department of Women’s and Children’s Health, Clinical Psychology in Healthcare, Uppsala university, Uppsala, Sweden

**Keywords:** eHealth, Palliative care, Patient, Literature review, Nursing

## Abstract

**Background:**

With a growing world population, a longer life expectancy, and more deaths due to chronic diseases, the need for palliative care is increasing. Palliative care aims to alleviate suffering and to promote well-being for patients with progressive, incurable disease or injury. E-Health entails using of information and communication technology for healthcare provision. It is unclear to how patients experience use of eHealth technology within palliative care.

**Methods:**

The aim of this study was to describe patients’ experiences of eHealth in palliative care. A systematic integrative review was performed using six databases: Cinahl Complete; MEDLINE; PubMed; Psychology and Behavioral Sciences Collection; Nursing and Allied Health; and PsycINFO. Twelve studies met the inclusion criteria of adult patients in palliative care, English language, published 2014–2019: comprising 397 patients. Six studies were from European countries, four from North America, one from South America and one from Oceania. Seven were feasibility or pilot studies.

**Results:**

The findings are synthesized in the main theme: *E-health applications – promoting communication on patients’ and families’ terms*, and three sub- themes: usability and feasibility of eHealth applications; symptom control and individualized care; and use of eHealth applications increased sense of security and patient safety. Patients’ experiences were that eHealth promoted individualized care, sense of security, better symptom management and participation in care. Communication was facilitated by the inherent flexibility provided by technology.

**Conclusions:**

E-Health applications seem promising in promoting equal, individualized care, and may be a tool to endorse accessibility and patient participation in palliative care settings. Indications are that eHealth communication resulted in patients and families receiving more information, which contributed to experiences of patient safety and feelings of security. At organizational and societal levels, eHealth may contribute to sustainable development and more efficient use of resources.

## Background

With a growing world population, a longer life expectancy, and more deaths due to chronic diseases, the need for palliative care is increasing [1, 2]. Palliative care aims to alleviate suffering and to promote well-being and quality of life for patients with progressive, incurable disease or injury. This includes taking physical, psychological, social and existential needs into account [3, 4], and further to provide family support [5]. Combining healthcare professionals in teams is suggested both to offer support and to allow meeting complex needs [6], so that patients may to continue living as they prefer until death. Communication is a key concept in palliative care [7], and an important dimension of patients’ experiences of care [8]. Perceptions of care quality and patients’ well-being are affected by possibilities for inclusive communication [9, 10]. Since global access to palliative care varies greatly [2, 11], and to facilitate patients’ being heard and cared for, exploring alternative approaches for patients to communicate their needs and preferences to healthcare professionals is crucial [12].

E-Health involves the use of information and communication technology (ICT) to provide care, and to transmit health information through the Internet and related technologies [9, 13], irrespective of distance. Other commonly used words are telemedicine and telehealth [14, 15]. Ideally, eHealth promotes patient involvement and participation in care, improves quality of care, and increases access to care while maintaining cost effectiveness [1, 13], especially in remote locations [16]. Other benefits are convenience, reduced travel time and reduced risk of infections. In a qualitative study, healthcare professionals express concerns regarding eHealth use in palliative care, both regarding technological issues and possibilities for creating a caring relationship through digital media [17]. The authors conclude that eHealth may be useful as a complement to regular face-to-face care. These findings are confirmed by a literature review investigating use of video consultations in palliative care [18], emphasizing improved communication, usability for diverse groups of patients and that relatives were included. However, using eHealth would also mean handling patient data and confidential information remotely, which highlights issues regarding storing and protecting confidential information [16]. Privacy and security issues were identified concerns alongside the fact that economic implications are unclear [18]. In another study, healthcare professionals expressed fear that eHealth would replace human contact and interaction [19]. Identified barriers to eHealth in Africa are access to technology, like modern mobile phones, and infrastructure surrounding internet and digital applications. In a literature review highlighting eHealth technologies in palliative care, applications for eHealth were charted, including analysis of strengths, weaknesses, opportunities and threats of the technology [20]. Findings indicate that eHealth is an emerging field with potential to improve comfort and quality of life, however, the validity of applications is rarely discussed and respecting patient preferences for forms of care requires further attention. A scoping review underscores that eHealth applications seem feasible, however, that telehealth in relation to burdensome symptoms and wellbeing warrants further investigation, together with studies of effectiveness in eHealth interventions [21].

**Fig. 1:**
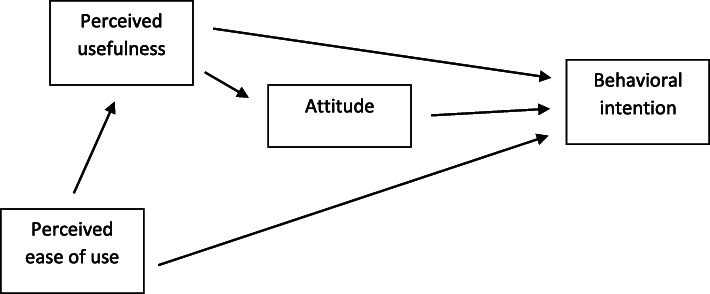
Overview of the Technology Acceptance Model (TAM)

The Technology Acceptance Model (TAM) explains how a user accepts the use of technology [22], and has been used in studies regarding eHealth application [23–25]. The goal of TAM is to gain an understanding and explanation of the user’s behavior and use of technology, where usability and ease of use indicate the user’s acceptance of the technology, see Fig. 1.

Other important factors are the utility and quality of the technique [22, 26], and that technology is adapted and meets needs [25]. There is interest in using quality-of-life instruments within eHealth in palliative care, as a foundation for reinforcing person-centered and participatory care [27, 28], however, a challenge remains in adapting ICT to patients’ individual resources and needs [12].

The United Nations (UN) Sustainable Development Agenda [29] emphasizes modern and effective care for all, and promotes development to create conditions for all people to attain their fundamental rights to health and well-being. Innovation and technological advances are crucial to finding sustainable solutions to both economic and environmental challenges, that in turn can contribute to efficient and equitable use of resources. Healthcare services and ideals are shifting towards a view of patients as proactive, well-informed, collaborative partners in healthcare provision [30], and this seems to align with patient preferences [5, 31]. Effectiveness increases if patients are involved in the process of their own care [1]. E-Health can be an effective way of supporting communication between patient and healthcare providers, and the technology to do so exists [20], but the evidence regarding patients’ experiences using ICT or eHealth applications within palliative care is scattered and unclear [9].

## Methods

The aim of the study was to describe the evidence regarding patients’ experiences of eHealth in palliative care.

### Design

An integrative review was chosen to allow inclusion of both experimental and non-experimental research, aiming to provide broad view of the phenomenon of eHealth, and to investigate the available evidence [32]. The study was informed by the outlined steps of Whittmore and Knafl [32], namely problem identification, literature search, data evaluation, data analysis and presentation.

### Problem identification and literature search

To pinpoint the problem, initially broad and general searches were performed to identify the variables of interest. The three areas *palliative care*, *technology* and *experiences* emerged as core concepts relevant to the aim. Systematic searches were performed in the following databases: CINAHL Complete, MEDLINE (full text), Nursing and Allied Health database, PsycINFO, Psychology and Behavioral Science Collection, and PubMed using the key words *telemedicine, patients and palliative care,* identified as relevant Medical Subject Headings (MeSH). Free text words added to the search were attitude, communication, experience, qualitative, discourse and views, see Table 1.

**Table 1:** Inclusion and exclusion criteria

Criteria	Inclusion	Exclusion
Types of studies	Qualitative, quantitative, and mixed methods original studies on the phenomenon and with explicit ethical considerations published in peer-reviewed journals	Letters, comments, conference abstracts, editorials, doctoral thesis, or any type of review
Period	January 1, 2014 until March 3, 2019	Before January 1, 2014, and after March 3, 2019
Languages	English and Swedish	All other languages
Type of participants	Patients in a palliative care trajectory regard-less of diagnosis, aged 18 years or older	Patients who are not in a palliative care trajectory, patients younger than 18
Phenomenon of interest	Patients’ subjective and objective experiences of eHealth in palliative care	Health care professionals’ and families’ views on eHealth in palliative care

The inclusion criteria were peer-reviewed original studies describing patients’ experiences of eHealth, with the words: telemedicine, patients, and palliative care in either title or abstract, and with explicitly stated ethical considerations by article authors. Exclusion criteria were studies published before 2014, participants under age 18, literature reviews and studies in languages other than English or Swedish, see Table 2. The five-year limitation was decided on due to the rapidly evolving technological advances and to evaluate the latest evidence in the field [21]. Pediatric palliative care for persons under 18 is a new subspeciality with heterogenous services, making comparisons challenging [33]. Literature reviews comprise older articles and were therefore excluded. No geographic limitations were set.

**Table 2:** Search strategy

Databases	MeSH-term/free text word	Search and limitations
Cinahl Complete Medline with Full Text Nursing & Allied Health Database Psychology and Behavioral Sciences Collection PsycINFO PubMed	Telemedicine AND Patients AND Palliative Care OR Attitude OR Communication OR Palliative Care Experience OR Qualitative OR Discourse OR Views	Advanced search. English and Swedish. 20,140,101–20,190,303. Peer-reviewed. Find all my search terms. Abstract available. All genders, all adults, all geographic regions, all publication types.

**Fig. 2:**
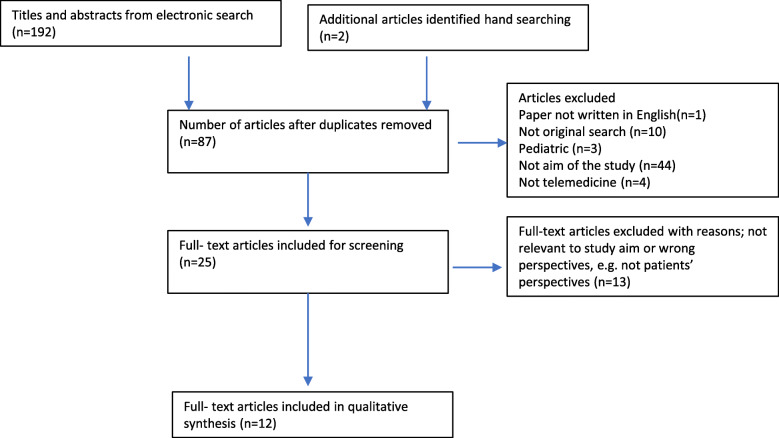
Search and inclusion process inspired by Moher et al. [34]

During the search process, all titles and abstracts were independently assessed by first and second author, each making an independent decision to whether to include or exclude for full-text screening. All articles included for full-text screening were similarly read and independently assessed by two persons. Consensus discussions ensued which determined articles to include in the review. The study inclusion/exclusion process is outlined according to the PRISMA structure as described by Moher [34], see Fig. 2.

### Data evaluation

All included articles were evaluated according to the Critical Appraisal Skills Program (CASP) depending on method used. Articles were not excluded if quality was found lacking, in line with Whittemore and Knafl’s recommendations [32], however, reflection on valid inferences aligned with scientific quality were performed throughout the research process. For details and results of the CASP evaluation, see Table 3.

**Table 3:** Quality assessment by CASP guidelines

Study authors	Q1. aims	Q2. method	Q3. design	Q4. recruitment	Q5. data collection	Q6. relationship	Q7. ethics	Q8. data analysis	Q9. findings	Q10. value
Benze et al.	Y	Y	Y	Y	Y	Y	Y	Y	Y	5
Bonsignore et al.	Y	Y	Y	Y	Y	Y	N	Y	Y	5
Cooley et al.	Y	Y	Y	Y	Y	Y	Y	Y	Y	5
Hennemann-Krause et al.	Y	Y	Y	Y	Y	N	Y	Y	Y	5
Hoek et al.	Y	Y	Y	Y	Y	Y	Y	Y	Y	5
Pinto et al.	Y	Y	Y	Y	Y	Y	Y	Y	Y	5
Tieman et al.	Y	Y	Y	Y	Y	Y	Y	Y	Y	5
Timmerman et al.	Y	Y	Y	N	Y	N	Y	Y	Y	5
van Gurp et al.	Y	Y	Y	Y	Y	N	Y	Y	Y	5
Vitacca et al.	Y	Y	N	Y	Y	Y	Y	Y	Y	5

Y – yes, N- nor or unclear; Q10 values rated from 1 to 5, with 5 high

### Data analysis

A core in the integrative review, as described by Whittemore and Knafl [32], is the qualitative, iterative nature. As suggested in the data reduction and data display phases, primary data were analyzed and arranged, coded, categorized and summarized in a matrix. This process started with a thorough reading and re-reading of the included articles, performed independently by two persons. Each person extracted data relevant to the aim and pasted into a matrix, initially assessing each article by itself, and later discussing the findings. In the data comparison phase, patterns and commonalities were noted, grouped together and contrasted in line with shifting perspectives to allow critical analysis of data. A thorough and impartial interpretation of data, promotes innovative synthesis of evidence [32], one goal of the data analysis step. This process of shifting perspectives and noting commonalities and patterns resulted in the creation of a main theme: *E-health applications – promoting communication on patients’ and families’ terms,* and three sub-themes. The themes were created based on groupings of findings, aiming to summarize the synthesis, and putting it into words that conveyed the underlying meaning, to present in a useful way to a wider audience [35].

## Results

This integrative literature review included 12 empirical studies [36–47], with a total of 397 patients. The qualitative studies comprised 187 patients and the quantitative studies 225 patients. Two studies used mixed methods, and the 15 participating patients were counted once even though they were part of two data collections. The patients’ ages ranged from 18 to 91 years, and studies were from Australia/New Zeeland [1], Brazil [1], Italy [1], Canada [1], the Netherlands [3], Portugal [1], Switzerland/Germany [1] and the United States [3], see Table 4 for details on the included studies. The care context in the studies was specialist palliative home care [36, 37, 41, 43–47], hospice or palliative care ward [39], hospitals or clinics supporting palliative patients at home [40, 42], and patients with ongoing cancer treatment [38]. Diagnoses of patients comprised a majority with advanced cancer [36, 38, 40–42, 45], one study with advanced chronic obstructive pulmonary disease (COPD), or a combination of diagnoses like cancer and other [39, 44], cancer, amyotrophic lateral sclerosis (ALS) and multiple sclerosis (MS) [43], cancer and COPD [46]. The majority of studies presented testing, feasibility, acceptability or pilot studies regarding eHealth interventions [36, 37, 39, 40, 42–45, 47, 48], with one RCT investigating care as usual to added telephone/video consultations [41], and one study investigated the effect of eHealth on patient-HCP relationships [46]. The eHealth applications pertained to symptom reporting and monitoring [36–38, 40, 41, 43–45, 47], using eHealth for flexible and increased communication between patients, families and HCP [36–40, 43, 44, 46, 47], patient guidance and medication adherence [36], psychosocial support [47], psychotherapy for young adults with cancer [42], and a physical fitness program [45].

**Table 4:** Matrix of included articles

Ref no	Author, year, country	Aim	Method, eHealth intervention	Sample	Main findings
[33]	Benze G, Nauck F, Alt-Epping B, Gianni G, Bauknecht T, Ettl J et al. (2017) Germany, Switzerland	The aim of the study was to test and assess whether a patient-reported-outcomes (PRO) - symptom estimation - via a newly developed smartphone application (MeQoL) is feasible for outpatients with advanced cancer	Prospective, uncontrolled, multi-centered, feasibility trial. Descriptive statistics. Smartphone application to monitor symptoms	Patients (*n* = 37) included in the study. Three patients were lost when three units disappeared in postal management. Adult patients in home care.	High patient satisfaction when using digital reporting via smartphone. Regular symptoms- and quality of life follow-up via an application shows significant clinical results. Patients experienced that the application could provide guidance and evaluation of given medication for pain. Smartphone application feasible for use in monitoring adherence to medication and facilitates patient guidance.
[34]	Bonsignore L, Bloom N, Steinhauser K, Nichols R, Allen T, Twaddle M, et al. (2018) USA	1) describe a telehealth palliative care program using the TapCloud remote patient monitoring application and videoconferencing; 2) evaluate the feasibility, usability, and acceptability of a telehealth system in palliative care; and 3) use a quality data assessment collection tool in addition to TapCloud ratings of symptom burden and hospice transitions	Mixed methods approach to assess feasibility, usability and acceptability; pilot study. Descriptive statistics, qualitative semi-structured interviews (*n* = 9). Digital platform for monitoring symptoms and video conferencing.	Patients (*n* = 101) in palliative care program in rural Western North Carolina with one or more life-limiting illnesses. Adults over the age of 18 with wireless network or 3G / 4G.	Increased patient satisfaction, decreases unnecessary health care, optimizes health care resources. Patients and the healthcare staff had predominantly positive experiences of the digital platform. Easy to use, does not take long to use. Acceptability, feasibility, and usability of telehealth and the TapCloud application demonstrated. Has potential to improve patient outcomes, and reduce unnecessary health care utilization, optimize resource allocation, and increase patient satisfaction.
[35]	Cooley ME, Nayak MM, Abrahm JL, Braun IM, Rabin MS, Brzozowski J, et al. (2017) USA	1) describe patient and caregiver perspectives for providing, processing, and managing symptoms and quality of life and (2) explore their perspectives about which components of decision support would be desirable to enhance communication with clinicians about symptoms and quality of life during cancer treatment.	Qualitative approach, nine focus groups. Thematic content analysis. Exploring preferences and suggestions for eHealth applications: symptoms and quality of life	Participants (*n* = 64), over 18 years, Eng/Spa language with ongoing or given cancer treatment in the last 6 months. Participants were paid.	Patients and caregivers described components of an eHealth system that might facilitate communication with clinicians and meet needs: (1) the ability to track symptoms over time, (2) access to Webbased information, including visual information, (3) DS that provides prompts for when to contact the clinicians to report their symptoms (ie, when pain severity reaches a “5” on a 0–10 scale) and/or an alerting system for clinicians, (4) peer support, and (5) access to medical records.
[36]	Guo Q, Cann B, McClement S, Thompson G, Chochinov HM. (2017) Canada	To explore the feasibility of introducing internet-based communication and information technologies for in-patients and their families, and to describe their experience in using this technology.	Cross-sectional study Descriptive and analytic statistics. Qualitative thematic analyses. Feasibility study of communication through internet using iPad or ThinkPad	Patients (*n* = 13) between the ages of 42–82 years in a palliative care ward, relatives (*n* = 38) and medical staff (*n* = 14). English speakers. Started with 95 patients and family members - drop out due to. - “not interested” and “inappropriate timing”.	Patient and close relatives used “keep in touch” KIT technology to communicate which made the patient feel better, became calmer, felt closer to relatives. The feasibility of offering internet-based communication and information technologies on palliative care in-patient units confirmed. Patients and families need to be provided appropriate technical support to ensure that the technology is used optimally to help them accomplish their goals.
[37]	Hennemann-Krause L, Lopes AJ, Araujo JA, Petersen EM, Nunes RA. (2015) Brazil	To examine telemedicine as a form of home and additional support for traditional outpatient care as a way to remotely monitor and manage the symptoms of patients with advanced cancer.	Prospective, longitudinal, qualitative, descriptive design with case studies. Interviews with patients. Using ICT for communication: web conferencing with care team and remote symptom assessments.	Patients (*n* = 12) older than 18 years with an advanced incurable cancer disease with access to data.	Telemedicine allowed greater access to the healthcare system, reduced the need to employ emergency services, improved assessment/control of symptoms, and provided greater orientation and confidence in the care given by family members through early and proactive interventions. Web conferencing proved to be a good adjuvant to home monitoring of symptoms, complementing in-person assistance.
[38]	Hoek PD, Schers HJ, Bronkhorst EM, Vissers KCP, Hasselaar JGJ. (2017) The Netherlands	To determine whether weekly teleconsultations from a hospital-based specialist palliative care consultation team (SPCT) improved patient-experienced symptom burden compared to “care as usual”. Secondary objectives were to determine the effects of these teleconsultations on unmet palliative care needs, continuity	Randomized controlled trial for 12 weeks. Primary outcome patient-experiences symptom burden comparing care as usual with scheduled telephone meetings. Teleconsultation with speaker and camera using iPad technology.	Home healthcare patients with cancer (*n* = 74). Adults over 18 years included. Patients completing study (*n* = 32).	Patients who received weekly contact consultation for the usual palliative care experienced increased / worse symptoms (regarding anxiety and depression) compared to other home health care patients. The number of unmet needs, experienced continuity of care, and reported hospital admissions did not differ between groups.
[39]	Melton L, Brewer B, Kolva E, Joshi T, Bunch M. (2017) USA	Investigating whether E-health use can be a way for young adults to meet to receive psychotherapy support in groups.	Questions online about the technology and its possibilities, satisfaction, as well as qualitative questions were asked. Telemedicine offering psychotherapy in web conferencing format using iPad technology.	Young adults (*n* = 8), 18–40 years, with cancer, from Colorado. English speakers. All participants had wireless internet as well as the habit of using an iPad or computer. One participant did not complete the questions.	Patients experienced an increased sense of belonging and satisfaction through group video conferencing. Available- warmth and continuity increased and the geographical differences in care reduced. The modern format increased access to care across a geographically diverse population, reducing health disparities between rural and urban communities.
[40]	Pinto S, Almeida F, Caldeira S, Martins JC. (2017) Portugal	To introduce a web-based application to monitor patients’ well-being in palliative care. Usability and acceptability studied.	Pilot study testing feasibility and acceptability in developed app for reporting symptoms and sending messages. Web-based application to monitor comfort and report symptoms.	Patients (n = 7) with cancer, ALS and MS, in palliative home care. Participants older or equal to 18 years with illness that will shorten their lives. Lost 2 people.	The patients experienced a high level of satisfaction. By having the care “kept track” of them, the application was very useful and easy to use. All patients gave high rating on 10 rating scale. Prototype feasible and acceptable for use. Needs further testing on larger scale.
[41]	Tieman JJ, Swetenham K, Morgan DD, To TH, Currow DC. (2016) Australia, New Zeeland	The study investigates the use of E-health in palliative care for patients, caregivers and clinics.	Prospective cohort study, of a telehealth-based intervention for community-based patients of a specialist palliative care service. Descriptive statistics, evaluation. Video-conferencing, reporting symptoms and accessing information online, using iPad technology.	Patients (*n* = 43) over 18, in home palliative care, able to manage computers, English speaking. Patients excluded if in bed more than 50% of the time.	The trial showed that patients and carers, including patients over 80 years, could manage the technology and provide data that would otherwise not have been available to the palliative care services. Self-reported data entered by patients and carers did identify changes in performance state and in symptom distress triggering alerts to the service provider. Scheduled videocall contacts and contacts made in response to triggers led to changes in care.
[42]	Timmerman JG, Tonis TM, Dekker-van Weering MG, Stuiver MM, Wouters MW, van Harten WH, et al. (2016) The Netherlands	To develop a multimodal application aimed at improving rehabilitation and physical activity after lung cancer surgery in close collaboration with healthcare professionals, and to evaluate the usefulness	Evaluation of co-creation and usefulness through semi-structured interviews with patients and healthcare professionals. Focus groups and scenarios for views of the technology. E-health application for symptom monitoring and physical fitness program.	Patients (*n* = 12) with lung cancer and healthcare professionals (*n* = 6). Both patients ‘and carers’ perspectives. Patients older than 18 years.	Both nursing staff and patient positive about using e-health applications and consultation. Patients experienced reduced uncertainty around perceived symptoms and increased sense of self-control and access to advice. A telehealthcare application that facilitates symptom monitoring and physical fitness training is considered a useful tool to further improve recovery following surgery of resected lung cancer (LC) patients. Involvement of end users in the design process appears to be necessary to optimize chances of adoption, compliance and implementation of telemedicine.
[43]	van Gurp J, van Selm M, Vissers K, van Leeuwen E, Hasselaar J. (2015) The Netherlands	To investigate how consultation via e-health can affect the relationship between patients in palliative home care and specialists in palliative care.	Qualitative, longitudinal study. Semi-structured interviews, open interviews and observations. Teleconsultation through video conference, iPad or computer.	Patients (*n* = 18) were included in the study, cancer (*n* = 16) and COPD (*n* = 2). Family members and caregivers were interviewed. Adults between 24 and 85 years. Patient drop out (*n* = 2), dissatisfied (*n* = 1), moved to hospice (*n* = 2) and asked for euthanasia (*n* = 1).	E-health consultation is appropriate for palliative care in the home. The consultation can also facilitate the contact between patients and healthcare staff, building relationships and improving quality of care. An implementation guide for e-health is described.
[44]	Vitacca M, Comini L, Tabaglio E, Platto B, Gazzi L. (2019) Italy	To test the feasibility of, and patient satisfaction with, an advanced care plan for severe COPD patients followed by tele-assistance at home for six months that focused on monitoring patient’s palliative topics through a dedicated checklist.	Telephone support by a specialist physician in Palliative care and then structured telephone calls by a nurse once a week about the patient’s clinical status and monthly about the patient’s needs with the help of a checklist. The study went on for 6 months. Qualitative analysis Telephone calls, weekly, for psychosocial support and symptom reporting.	Patients (*n* = 10) with severe COPD with less than one year left to live. Adults At least three of the following: 1) FEV (Forced Expiratory Volume < 30%; 2) at least 3 hospital admissions in the last 12 months 3) > 5 years of long-term oxygen therapy; 4) shortness of breath and signs and symptoms of heart failure.	The patient experienced reduced anxiety during the conversation. The patients described even bad days with negative emotions and deterioration in their illness. All patients expressed a high level of satisfaction with the support. The feasibility and benefits (more communication between hospital staff and patients and optimized management of symptoms) of offering a PC intervention to patients confirmed.

The results are presented in the main theme: *E-health applications – promoting communication on patients’ and families’ terms*, and through three sub- themes: (1) usability and feasibility of eHealth applications; (2) symptom control and individualized care promoted through eHealth applications; (3) use of eHealth applications increased sense of security and patient safety.

### E-health applications - promoting communication on patients’ and families’ terms

Throughout the literature review, findings were that eHealth applications generated multiple arenas for communication on patients’ and families’ own terms, and in their own time. In all the included studies, patients’ experiences were predominantly positive for eHealth applications and communication [36–47]. Eleven studies described patients’ practical experiences of using various technological tools to communicate digitally with their caregivers [36, 37, 39–47]. In the twelfth study, patients described wanting technical communication aids both to receive and to provide information digitally, and thus facilitate communication with healthcare professionals [38].

### Usability and feasibility of eHealth applications

Through technology, new opportunities and arenas for meetings were made possible, as an addition to the more common traditional face to face care meetings or conferences. Several studies described that patients perceived various technical communication aids as user-friendly and feasible [36, 37, 39–47]. An example of user flexibility, was an app that could be used in the way individual patients or families wanted, either on a mobile phone, a computer or a tablet [37, 39, 43–45]. Communicating via video link also worked well and was found convenient [37, 39–43, 46]. Patients experienced benefits of videoconferencing as compared to telephone consultations, since the added visual dimension provided information about body language and emotions [39, 40, 44]. The majority of participants in two studies were very satisfied with video meetings, and even preferred them before physical meetings [42, 44]. Another advantage of videoconferencing, compared to telephone consultation, was that how patients were feeling quickly became apparent, which was found convenient by patients since they did not need to explain themselves [46]. The added visual information was desired and considered useful [38]. However, contradictory findings were found in two studies; one where patients favored the personal encounter and found that a deeper personal connection could not be achieved through eHealth technology [37], and in another study, some patients and families felt that they were satisfied with telephone calls and information by letter, since they found new technology difficult [38].

One important factor for adopting eHealth applications was the attitude and enthusiasm of the healthcare professionals (HCPs) [44, 45]. If the HCPs were not excited and motivated to use the new technology, neither was it used by patients or families. None of the studies indicated the use of eHealth technology was restricted by high age, and a mix of ages used eHealth technology in the included studies [36–47]. Older people were able to manage the technology well, but sometimes needed additional support at the start [36, 37, 44]. In case of technical problems, these were solved through support from HCPs or through help from family members [39, 40, 45].

### Symptom control and individualized care promoted through eHealth

Various eHealth applications enabled patients to participate in and govern their own care, for example by self-reporting symptoms and needs [36–41, 43–45, 47]. The possibility of sending text messages to HCPs, for example to give notice that medication needed refilling, was perceived by patients as a well-functioning alternative for communication. Information from validated instruments describing physical, mental, social and existential symptoms, as well as quality of life, was sent to HCPs using eHealth technology, and was subsequently used for making care and treatment decisions [36–41, 43, 44, 47]. Participants experienced a high degree of satisfaction when videoconferencing was conducted to address concerns raised by previously submitted information [42, 46]. Patients found that communication was improved through eHealth applications, since instant feedback and help were provided as a result of the submitted, self-reported symptoms [36–38, 40, 44]. This enabled individualized care according to patients’ and families’ needs. Patients wanted to register and monitor symptoms over time since it was found valuable method to be able to manage their symptoms themselves [38, 45], and patients were highly motivated to be involved in their own care [36, 42, 47]. Studies also showed that automatic reminders to take their medication could be of benefit to the patient [36–38, 43]. Improved symptom control empowered patients to remain in their homes until the end of life [40]. However, in one study, some patients experienced increased symptoms, but the nocebo effect could not be rejected [41].

Care was perceived as more accessible and patients described increased access to care [36, 37, 40, 42–47]. Communication and consultation with HCPs through eHealth applications aligned with patients’ daily lives, since it enabled patients to determine the time of contact themselves [36, 43, 46]. Another finding was that using digital consultations could condense health care meetings and make them more efficient [37, 40, 42, 44, 46]. eHealth application could be tailored to patients’ needs, thus enabling individualized care [37, 39, 42, 43, 45–47].

Through eHealth technology, patients were able to maintain their social relationships and contacts in everyday life, since they were able to be cared for at home [39, 40, 42, 46]. With the help of eHealth, patients could continue to participate in social activities like before the illness [39, 42]. Despite long distances, it was possible for patients to receive care through eHealth applications, in a convenient manner, without spending time nor funds for travelling [40, 42]. During treatments that resulted in a low immune response, eHealth was a good way to meet, participate and interact with peers to feel support [42]. Being able to participate in both private and social contexts, as well as in caring and supportive situations, despite troublesome symptoms and severe illness, was perceived as positive and provided a sense of connection to life and living [36, 39, 40, 42, 45].

### Use of eHealth applications increased patient safety and sense of security

Several studies described how patients felt that information, guidance and advice could be transmitted safely through various eHealth applications [36–38, 40, 41, 43–45, 47]. An application enabled the healthcare staff to see the text in their language even though the patient wrote it in another language [37]. Patients stated that they received a guarantee that the information was forwarded to the intended recipient, and further that they experienced a faster response [37, 46]. Patients also expressed that they did not want to interfere, and therefore found eHealth applications less intrusive since HCPs monitor them at their own convenience [38]. Thus, patients did not hesitate to contact HCPs through the application. Patients admitted to palliative care also described how difficult it was to remember and understand all the information provided about diagnosis, treatment and potential symptoms and how easy it was to forget the information [38]. Patients described how they relied on the family members and therefore wished to share information with health care professionals, friends and family through the eHealth application [38, 39, 42]. E-health consultation provided a sense of security, relief and accessibility; for example, reminders to take medication [36–38, 43], facilitating renewal of prescriptions [37], increased access to care and prolonged care meetings [37, 40, 43], feeling better involved in own care [40], quicker response time to queries [37, 45], and that information, advice, and guidance could be safely delivered due to eHealth applications [37, 47].

Patients described how the eHealth applications provided increased opportunities and circumstances to feel secure [37, 39, 40, 42–47]. In a study where patients had not tried the technology, patients anticipated that use of eHealth applications could mean feeling safe at home, since call for help and a follow-up visit was facilitated through the technology [38]. Increased access to care resulted in increased confidence in the care, which may have contributed to reduced emergency hospital admissions [40, 46], since patients felt supported at home. Patients using different eHealth applications experienced security and increased well-being, as well as peace of mind and feeling at ease [37, 39, 40, 43, 45–47]. A negative finding was that the integrity of the patient could be jeopardized since other persons unannounced could enter a room during an ongoing meeting [44, 46]. This resulted in patients experiencing a sense of intrusion.

## Discussion

The results of this integrative review highlight potential for eHealth applications since patients’ experiences were that eHealth applications promoted and improved communication with healthcare professionals in palliative care. Through eHealth applications, these patients had access to a convenient information- and contact channel on their own terms, and furthermore, felt empowered to participate and govern their own care, which resulted in experiences of security. In the twelve studies in this review, most patients found the eHealth applications easy to use and helpful in managing their daily lives with life-threatening illness. However, the included studies comprised testing, feasibility, and acceptability studies on small populations of patients in palliative care. More robust, controlled studies are needed to establish generalizability to larger populations, and to produce evidence to guide future implementation efforts regarding eHealth in palliative care.

Ability and willingness to accept new ICT, ease of use, and usefulness has been explored using the TAM model [22]. The model has been widely used to predict and explain users’ reactions to new information technology, and has become a gold standard [49]. An early idea with using the model was to encourage use of ICT and to think of ways to increase modern advances [22], including healthcare settings. However, technology and palliative care may seem polar opposites since technology lacks human touch. Palliative care is described as inherently built on communication and interaction between patients, families and healthcare professionals [7, 20], and as such demands sensitivity and flexibility to promote understanding of experiences and goals of care in alignment with patients’ wishes [50, 51]. Contrary to this, the results of this review indicate that use of eHealth applications in palliative care may provide feasible alternatives without violating palliative care standards. Some patients even found that eHealth improved communication and connectivity with the healthcare providers. Thinking of the palliative care dimensions, physical, psychological, social, and spiritual [4], theses studies did not explicitly address eHealth in relation to the dimensions. However, symptom monitoring (*physical*) and quality of life was studied [36–38, 40, 43–45, 47], communication (*social*) and consultations [39–41, 46], psychotherapy and psychosocial support (*psychological/existential*) [42, 47], and physical fitness [45], comprising palliative care dimension content. The studies do seem to focus on physical challenges and eHealth as a tool for communication, while social, psychosocial, and existential aspects fall behind.

The potential benefits of eHealth can be increased access to care, increased convenience, reduced travel time and reduced risk of infections [52, 53]. These aspects are reflected in the results of this review, and in line with previous findings from Pinto et al. [20]. Communication between patient, close relatives and team members was facilitated through eHealth, and patients’ experienced increased access to care since physical distance was not an issue. Patients found that the use of video consultation, strengthened communication since visual features are included, also described in other studies [53, 54]. Another benefit found in our review was that despite life-threatening illness and being close to the end of life, patients could take an active role and govern their own care if they want to, thus promoting individualized care. The use of technology has potential to increase access to healthcare and possibly provide savings [53], however, implications of economics and effectiveness have not yet been established [18, 21]. Allsop et al. [12] describe that patients in palliative care are willing to try new technologies as part of their care. In our review, benefits of technology mentioned were that it could be used for language translations, response time could be quicker, and it was perceived as less intrusive than a home visit. These benefits align with dimensions of usefulness as described in the TAM model [55].

The result indicate that eHealth applications may promote participation, security and safe care. One way to participate was self-reporting symptoms through mobile phones, tablets or internet, which also has been described by Cooley et al. [48]; and facilitating symptom reporting in this manner consequently resulted in faster access to care [48, 56]. For nurses, using validated questionnaires in combination with eHealth applications, may be one way forward to enhance quality of care on the terms of the individual patient. This approach could also promote patients’ motivation and adherence to monitoring symptoms and governing their own care. Video meetings have had positive effects on patients’ knowledge and provided social support for people with chronic illness [54], which agrees with the results of this study. However, safeguarding the integrity of patients in the context of video meetings is crucial to maintain a sense of security. Also, it is imperative that registered nurses keep in mind those who do not have or use eHealth applications, to ensure that patients have access to equal care and treatment, where needs are met, and strive to counteract exclusion [57], since everyone will not want or be able to use eHealth applications. There also seems to be a risk that when submitting data for interpretation by health care professionals, the act in itself may generate a sense of security, which unfortunately may result in ignoring warning signals and waiting for an intervention. In a time of change and implementation of new technology, it is imperative that responsibilities surrounding self-monitoring are specified and that there is a clear plan for how to respond to ‘red flags’ in the system. With this taken into account, using eHealth applications within palliative care, seems aligned with the philosophy of a holistic view of patients [4] where security, participation and patient safety are pivotal [58]. This also seems reasonable considering that the technology is easily available for many countries and regions in our time.

**Fig. 3:**
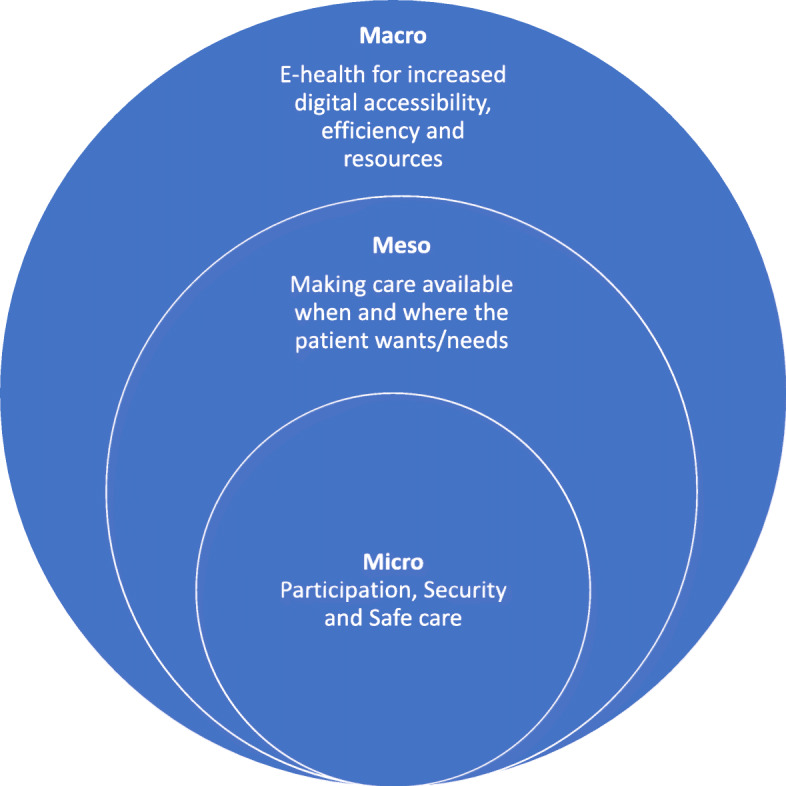
Clinical microsystems as described by Nelson et al. [59]

According to Nelson et al. [59], health care systems can be understood on multiple levels. The microsystem is the immediate vicinity around the patient, and in this system, values ​​are created for the patient. Depending on patient’s needs, the structure of the microsystem changes over time. The goal of the microsystem is to create the best possible value in care, relative to needs, for the patient. The mesosystem, or community level, describes interactions between units, while the macro system (society), provides support and structure to facilitate the work of the micro systems [59]. The findings of this review may be reflected on from a health systems perspective, see Fig. 3.

When patient values, like participation and security, ​​are established at the micro level, this may be understood at meso- and macro levels as well. At meso levels, eHealth may provide better chances for care availability on patients’ terms, and at macro levels, societies have opportunities to generate and provide infrastructure, including resources to implement eHealth applications. Developing and implementing eHealth applications for patients may also be a feasible way to promote the UN Agenda 2030 [29] sustainability goal 3, *good health and well-being*, and goal 9, *industry, innovation and infrastructure*, to reduce inequalities for a better and more sustainable future for all. Or as the case may be in palliative care, meeting physical, psychological, social and existential needs when time is limited and death is near [7].

Finally, it is our belief that eHealth should be seen as a complement to the face-to-face meetings and not as a substitute or replacement for physical meetings. Human contact and human interactions are inherent to nursing [60], and relationships between patients and health care professionals are important in palliative care [61]. However, that does not negate the tentative positive experiences of eHealth applications found in this review. A thought before the study, was that perhaps patients in palliative care would be too seriously ill to manage or want to use eHealth applications, and that older patients would find it difficult to use. However, the results indicate potential for patients in palliative care, regardless of age and illness, to use eHealth in addition to traditional care. There will be possibilities and challenges with eHealth applications, for example with regards to patient integrity [18, 20, 21], and there is reason to carefully consider the knowledge base for implementation of eHealth in palliative care. This review focused on patients’ experiences of eHealth in palliative care, and accordingly, it is challenging to state recommendations when considering implementation of eHealth technologies in palliative care. More robust studies with an element of randomization in accordance with the Medical Research Council guidelines [62], including evaluation of minimally clinically important differences [63], are called for to follow up feasibility and acceptability studies. With a holistic view of patients’ needs and well- being, eHealth may contribute to increased value ​​on several levels (micro, meso, macro) for patients, palliative care services, and health care systems.

### Limitations

There are a number of limitations to consider in this study. A plethora of different terms are used within the area of eHealth, and it is possible that some terms were missed in our search strategies. Patients in various palliative care settings have been included in this review, however, without classifying interventions, diagnoses, or care context. Language restrictions may have resulted in information bias for studies published in languages other than English. In this review, we aimed to describe the latest evidence regarding eHealth in palliative care. The search strategy was focused on research published from 2014 using the term palliative care. A more comprehensive review of literature using wider terminology may generate other articles of interest. An integrative review comprises a qualitative approach, and synthesis of various types of research findings may be challenging. For credibility and trustworthiness, we have strived for transparency in reporting methods and found that the themes were consistent throughout the included studies. From a global perspective, resources are unevenly distributed and implementing eHealth in countries and regions with low income may be both impossible and unrealistic. However, exploring local adaptations may be one way forward in investigating eHealth interventions in regions with differing resources and circumstances.

## Conclusions

E-Health applications seem promising in promoting equal, individualized care, and may be a tool to endorse accessibility and patient participation in palliative care settings. Furthermore, indications are that eHealth communication resulted in patients and families receiving more information, which contributed experiences of patient safety and feelings of security. At organizational and societal levels, eHealth may contribute to sustainable development and more efficient use of resources in palliative care.

## Data Availability

The datasets used and/or analyzed during the current study are available from the corresponding author on reasonable request.

## Abbreviations

CASP: Critical appraisal skills program
HCP: Health care professional
ICT: Information and communication technology
TAM: Technology acceptance model
UN: United Nations
